# New Horizons in Carbohydrate Research and Application for Endurance Athletes

**DOI:** 10.1007/s40279-022-01757-1

**Published:** 2022-09-29

**Authors:** Tim Podlogar, Gareth A. Wallis

**Affiliations:** School of Sport, Exercise and Rehabilitation Sciences, College of Life and Environmental Sciences, University of Birmingham, Birmingham, UK

## Abstract

The importance of carbohydrate as a fuel source for exercise and athletic performance is well established. Equally well developed are dietary carbohydrate intake guidelines for endurance athletes seeking to optimize their performance. This narrative review provides a contemporary perspective on research into the role of, and application of, carbohydrate in the diet of endurance athletes. The review discusses how recommendations could become increasingly refined and what future research would further our understanding of how to optimize dietary carbohydrate intake to positively impact endurance performance. High carbohydrate availability for prolonged intense exercise and competition performance remains a priority. Recent advances have been made on the recommended type and quantity of carbohydrates to be ingested before, during and after intense exercise bouts. Whilst reducing carbohydrate availability around selected exercise bouts to augment metabolic adaptations to training is now widely recommended, a contemporary view of the so-called *train-low* approach based on the totality of the current evidence suggests limited utility for enhancing performance benefits from training. Nonetheless, such studies have focused importance on periodizing carbohydrate intake based on, among other factors, the goal and demand of training or competition. This calls for a much more personalized approach to carbohydrate recommendations that could be further supported through future research and technological innovation (e.g., continuous glucose monitoring). Despite more than a century of investigations into carbohydrate nutrition, exercise metabolism and endurance performance, there are numerous new important discoveries, both from an applied and mechanistic perspective, on the horizon.

## Key Points

**Table:** 

Athletes should apply a periodized approach to nutrition to ensure dietary carbohydrate intake matches the carbohydrate demand of training or competition.
Contemporary sports nutrition guidelines allow for a certain degree of tailoring of carbohydrate intake to support a periodized approach.
Future research integrating individual athlete physiological data with sports nutrition knowledge will enable increasingly personalized ways to meet dietary carbohydrate intake goals for athletes.

## Introduction

It is now generally accepted that carbohydrates are perhaps the most important energy substrate for elite performance [1, 2]. However, this has not always been the case. At the start of the modern Olympic Games in 1896, protein was thought to be the most important energy source for athletes [3]. Research showing the importance of carbohydrates for performance began to emerge as early as the 1920s [4]. Further seminal work conducted in the 1960s [5], as reviewed elsewhere [3, 6], clarified the importance of dietary carbohydrates for exercise and in particular the role of muscle glycogen in endurance performance. However, it was only at the Montreal Olympic Games in 1976 that an appreciation of the importance of dietary carbohydrates among athletes and coaches started to emerge [3]. Together with other advances made towards improving athletic performance, such as improved equipment and training methodology, researchers continued to study carbohydrate metabolism to better understand the mechanisms of how dietary carbohydrates improve performance, promote recovery and/or prevent fatigue, as well as researching strategies to optimize carbohydrate availability in athletes [7–10]. Over this period, understanding of the actions of dietary carbohydrates on exercise metabolism and performance has increased substantially and, in concert, nutritional recommendations for athletes have developed and continue to evolve to reflect contemporary knowledge and practice. Nonetheless, scientific unknowns remain that, if addressed, could provide athletes with even more detailed and tailored recommendations to support them to reach their athletic potentials.

The purpose of this narrative review is to present contemporary perspectives and discuss areas for future research and application regarding the role of dietary carbohydrates for endurance athletes. The article is structured around three main sections: (1) the importance of carbohydrates for competition and high-intensity training sessions; (2) carbohydrates and training adaptation; and (3) personalizing carbohydrate intake. The article concludes by highlighting selected areas that, with further understanding, could help to further optimize the role of carbohydrates in endurance athletes’ diet (Table 1).

**Table 1:** Suggested areas for investigation to enhance understanding of the role of carbohydrates in the diet of athletes

Research topic	Research question	Practical relevance
Before intense workouts	How can glycogen storage be optimized when there is less than 24 h between 2 bouts of prolonged high-intensity exercise?	This is relevant for athletes competing day after day (e.g., cycling grand tours) or undertaking strenuous training sessions (e.g., training camps)
	To what extent can liver glycogen content be increased prior to exercise and is liver glycogen supercompensation possible?	This would advance our knowledge about liver glycogen—an important glycogen reservoir within the body
	Can ingesting multiple type monosaccharides (i.e., glucose with galactose and/or fructose) at breakfast help replenish liver glycogen quicker than glucose based carbohydrates?	Possible improvement of performance and/or exercise capacity due to higher glycogen availability; could influence incidence of hypoglycemia due to different intestinal absorption kinetics and subsequent metabolism
During intense exercise	How does carbohydrate ingestion during exercise affect compartmentalized muscle glycogen use (i.e., intermyofibrillar, intramyofibrillar or subsarcolemmal glycogen)?	Knowing this would provide further information on the mechanistic basis of carbohydrate ergogenicity
	Are exogenous carbohydrate oxidation rates during exercise absolute exercise intensity dependent?	We currently recommend athletes to ingest carbohydrate at certain rates without specific adjustments for energy turnover
	Can more than 60 g/h of exogenous glucose be oxidized if exercise intensity is sufficient and should carbohydrate recommendations during exercise be based on absolute exercise intensity and thus energy turnover rates?	Current knowledge is based on moderately trained athletes; exercise intensity and carbohydrate requirements are higher in elite athletes
	What is the role and what are the mechanisms of action of hydrogel forming carbohydrates in gastrointestinal comfort and exogenous carbohydrate oxidation rates?	Conflicting evidence exists regarding this form of delivery of carbohydrates during exercise
	What is the best strategy to offset higher total carbohydrate oxidation and lower exogenous carbohydrate oxidation rates in extreme environments (i.e., hypoxia and heat)?	Competitions often take place in stressful environmental conditions such as in the heat or in hypoxia. It is therefore important to optimize carbohydrate availability in those scenarios
After intense exercise	Would elite athletes benefit from higher short-term hourly carbohydrate ingestion rates as currently recommended 1.2 g·kg^−1^BM·h^−1^?	Current knowledge is based on moderately trained athletes whose muscle glycogen storage capacity is lower than in elite athletes
	When is it advisable to ingest a mixture of different monosaccharides (i.e., glucose, fructose and galactose) in recovery?	Relevant for athletes competing with short recovery times (< 24 h)
Carbohydrate periodization	Do low-carbohydrate availability training sessions improve training adaptations in a training program in which training volume is unrestricted (i.e., professional athletes) and are they thus more than just a time-efficient way of training?	Current evidence points towards this approach being efficient in athletes training up to 12 h per week, and it remains to be demonstrated whether it enhances adaptations when training volumes are higher
RED-S and overtraining	Is carbohydrate availability the most important factor in occurrence of RED-S and associated overtraining?	This would advance our understanding about RED-S and overtraining and provide better advice for prevention of these conditions
Personalization	What tools can be used to precisely prescribe carbohydrate intakes before/during/after exercise to improve performance and/or recovery?	While current carbohydrate guidelines offer a fair degree of personalization, knowing exact carbohydrate requirements (i.e., oxidation rates and endogenous carbohydrate availability) and exogenous carbohydrate oxidation limitations during exercise could lead to better individualization of carbohydrate intake during exercise
	What can be learnt from continuous blood glucose monitoring throughout the day and during exercise in athletes?	Better understanding of daily blood glucose profile in athletes could enable refined adjustment of carbohydrate availability in real time, resulting in optimizing recovery and/or performance

*BM* body mass, *RED-S* relative energy deficiency in sport

## Importance of Carbohydrates for Competition and High-Intensity Training Sessions

Sufficient energy availability from carbohydrates is required for athletes to sustain the demands of competition and recover for subsequent competitions [11]. Undertaking intense exercise sessions (i.e., competitions and high-intensity training sessions) places large demands on carbohydrates [12]. Additionally, the capacity to perform high-intensity sessions relies heavily on activation of carbohydrate metabolic pathways [13]. While energy for adenosine triphosphate (ATP) production can be derived both from fats and carbohydrates, it is carbohydrates that are primarily utilized at high exercise intensities [14, 15]. Evidence indicates that utilization of carbohydrates and not fats is crucial to sustain the exercise intensities observed in elite athletes [16, 17]. However, the importance of an ability to oxidize fats at high intensities should not be discounted [18], as a valid method to quantify its contribution to ATP production during exercise at high intensities is yet to be established [19]. Due to limited endogenous carbohydrate availability [20, 21] in endurance sports such as road cycling and cross-country skiing, energy requirements for races can exceed the capacity to store carbohydrates by more than 100% [22, 23]. Thus, strategies have been proposed to circumvent this issue, such as increasing carbohydrate storage before the start of competition [24]—also known as carbohydrate or glycogen loading, increasing exogenous carbohydrate availability by carbohydrate feeding during exercise [25] and/or reducing reliance on endogenously stored carbohydrates while increasing the utilization of fatty acids [26].

### Pre-competition

It has been known for decades that pre-exercise carbohydrate stores can influence exercise capacity [27]. The ability to store muscle glycogen increases with improved training status [28]. With training and dietary manipulations, muscle glycogen concentrations can increase to as much as 793 ± 170 mmol·kg^−1^ dry mass in highly trained populations [28]. While storage capacity is increased in highly trained populations, it appears that at the time of exhaustion, muscle glycogen concentrations are lower in untrained (i.e., 190 ± 90 mmol·kg^−1^ dry mass) than in trained populations (i.e., 280 ± 90 mmol·kg^−1^ dry mass) [28].

Recently, understanding of the underpinning mechanisms responsible for improved exercise capacity with elevated glycogen stores has improved. It has been demonstrated that glycogen loading increases exercise capacity by sparing of the intra-myofibrillar glycogen pool and that the intra-myofibrillar pool in type I muscle fibers is the best predictor of prolonged exercise capacity [29]. During high-intensity exercise efforts (e.g., sprints), the glycogen breakdown rate in type II fibers is higher than in type I fibers and this could indicate that glycogen content in that pool is more important at these intensities [30]; further work is required to see if this is the case. Mechanistically, impaired muscle function following muscle glycogen depletion appears to be due reduced Ca^2+^ release from the sarcoplasmic reticulum [29, 31].

As a result of the plethora of research demonstrating the importance of muscle glycogen availability, strategies have been devised on how to optimally stimulate muscle glycogen synthesis in the days leading up to competition, a strategy also known as carbohydrate or glycogen loading [1, 32]. To achieve muscle glycogen loading, athletes are recommended to consume a very high carbohydrate diet (i.e., 10–12 g·kg^−1^ body mass [BM] for 36–48 h before a competition [1, 2, 21]. However, it is common for athletes to undertake an exercise session in this time frame, during which some of the stored glycogen will be used. In addition, some sports events (e.g., cycling grand tours) consist of races taking place daily when there is less than 24 h available for recovery, and in these scenarios, optimization of carbohydrate intake in the post-exercise recovery period is paramount. Indeed, evidence indicates that maintaining high muscle glycogen concentration could be difficult in these circumstances [33]. More research is required to understand how to better sustain glycogen loading over multiple days, but most likely optimized recovery practices and in-race feeding strategies will represent the most effective ways to maintain adequate carbohydrate availability. In addition to this, more research is required to better understand how, and if it is possible to, affect skeletal muscle glycogen storage localization by diet manipulation, especially when time for carbohydrate ingestion is limited. A greater understanding of whether different types of carbohydrate affect the storage localization within muscle is also warranted.

While muscle glycogen depletion is more commonly associated with prolonged strenuous endurance exercise, even short but intense exercise bouts can result in significant reduction of muscle glycogen content [30, 34, 35]. Thus, one could assume that because of this, glycogen loading would be warranted. However, the evidence is not as conclusive, as glycogen loading before shorter duration events does not always translate into performance improvements [30, 36]. For instance, Sherman and colleagues showed that for runners undertaking a half-marathon, glycogen loading was of no benefit to performance [36]. Based on this, it is not currently recommended to perform glycogen loading for events shorter than 90 min [32]. It is important that carbohydrate availability is still sufficient, yet targeted carbohydrate loading is less warranted as compared to when strenuous exercise is to be performed for > 90 min. It must also be acknowledged that glycogen storage causes water retention and thus elevates athletes’ BM [37, 38]. This is especially pertinent for sports where increased BM could significantly hinder performance by reducing sustainable relative exercise intensity (e.g., W·kg^−1^) and/or reduce exercise economy [39, 40]. However, it is important to discern between functional BM (i.e., glycogen) and non-functional (i.e., excess body fat mass), as the utilization of the former during the event will cause a reduction of BM and could therefore only have a negative impact in the early parts of prolonged events.

Whether liver glycogen stores can also be super compensated remains to be established, as currently there is no clear evidence that this is possible [20]. Varying carbohydrate intake does indeed change fasting liver glycogen concentrations [41], and this shows that sufficient carbohydrate intake is required to start an exercise session with normal liver glycogen stores. However, whether glycogen stores could be increased to higher-than-normal levels remains to be established. It is often overlooked that if competition is to be performed in the morning after an overnight fast (or any period of prolonged fasting), liver glycogen stores may be compromised. Namely, in the postabsorptive phase (e.g., during the night) liver glycogen stores decrease [41]; so, in the morning they can be substantially depleted [42]. On the contrary, muscle glycogen stores are typically not affected by an overnight fasting period [42]. As numerous competitions start in the morning, it is pertinent that the meals after overnight fast are designed so that there is a focus on liver glycogen repletion. While this has not been directly assessed, it could be speculated that providing athletes with a mix of different types of monosaccharides would be beneficial. This suggestion is based on previous observations that combining glucose-based carbohydrates with either fructose or galactose offer benefits on liver glycogen synthesis over glucose-based carbohydrates only [43–45]. Indeed, a recent study found improved exercise capacity with a breakfast consisting of fructose-glucose-based carbohydrates as compared to glucose-based carbohydrates only [46].

It is expected with further research that the general guideline recommending 1–4 g·kg^−1^ of BM of carbohydrates as a pre-exercise meal [1, 2] will be updated with more granular recommendations based on the timing and the type of carbohydrates to be ingested. This would advance practical advice not only from the perspective of optimization of liver glycogen levels, but also from the perspective of preventing consequences of rebound hypoglycemia, which can occur in some athletes when exercise bouts are initiated close to a meal [47].

### During Competition

It is now well established that carbohydrate intake during exercise improves exercise performance/capacity [48]. Current nutritional guidelines for athletes advise to consume carbohydrates during exercise at different rates and in relation to the duration of exercise bouts, as will be discussed later [1, 2]. There are currently two proposed mechanisms of carbohydrate ergogenicity. Firstly, carbohydrates can be sensed in the oral cavity, causing an activation of certain brain regions, leading to stimulation of the central nervous system, as shown by improved performance by carbohydrate mouth rinsing [49–51]. Secondly, and most importantly, carbohydrates provide an additional fuel source for ATP formation during exercise. Carbohydrate ingestion during exercise maintains stable blood glucose levels over long exercise sessions [52] and maintains carbohydrate oxidation rates despite declining muscle glycogen stores so that ingested carbohydrates substitute endogenous carbohydrate stores [53, 54]. In addition to this, exogenous carbohydrates can spare or even completely suppress liver glycogen breakdown [55, 56]. While some studies have found sparing of muscle glycogen with carbohydrate supplementation during exercise [57, 58], most of the studies assessing whole muscle glycogen utilization did not see this effect [28] and a recently published study that evaluated different carbohydrate ingestion rates during cycling exercise did not observe sparing of muscle glycogen in a muscle fiber type-specific manner either [59]. However, more recent evidence indicating the importance of compartmentalized glycogen metabolism to muscle function opens new avenues for investigating the mechanistic basis of carbohydrate feeding during exercise [29].

It is recommended that athletes ingest up to 60 g·h^−1^ of rapidly oxidized carbohydrates such as glucose, glucose polymers and glucose-fructose mixes for exercise sessions that are up to 3 h long [1, 2, 60]. Galactose has typically not been recommended to be ingested during exercise due to a belief that it is not as readily oxidized [61, 62]. However, recent evidence demonstrates that at moderate dosages (i.e., 48 g·h^−1^), lactose (a molecule comprising glucose and galactose) can be as readily oxidized as sucrose (a molecule comprising glucose and fructose) while sparing endogenous carbohydrates [63]. It remains to be demonstrated directly, but these data indicate that oxidation of galactose during exercise is not limited when provided as lactose at moderate ingestion rates. Thus, lactose as a source of galactose and glucose can be ingested as an alternative carbohydrate source during exercise, at least in lactose-tolerant individuals.

The 60-g·h^−1^ recommendation is based on studies showing that when glucose-based carbohydrates are ingested during exercise, exogenous carbohydrate oxidation rates peak at 1–1.1 g·min^−1^, even if the ingestion rate is higher [25, 64, 65]. It is believed that at this ingestion rate, the sodium-glucose linked transporters (SGLT1) in the small intestine become saturated [25]. This notion is based on the observations that when fructose, whose absorption from the small intestine utilizes a different transporter (i.e., glucose transporter 5 [GLUT5]), is co-ingested with glucose, exogenous carbohydrate oxidation rates are elevated, and gastrointestinal issues are less prevalent [66–69]. As a result of this, athletes are recommended to ingest mixtures of glucose- and fructose-based carbohydrates when training or competition is longer than 2.5 h and when 60–90 g·h^−1^ of carbohydrates are ingested [1, 2, 60].

Most studies investigating combined ingestion of glucose-based carbohydrates and fructose utilized a 2:1 glucose:fructose ratio, and this has since become a standard recommendation [60]. However, a closer examination of the literature reveals that a ratio closer to unity (i.e., 1:0.8 glucose:fructose) is superior both from the perspective of oxidation efficiency (i.e., the fraction of ingested carbohydrates being oxidized) as well as gut comfort, and this occurs even at glucose ingestion rates below 60 g·h^−1^ when glucose absorption is thought not to be limiting [69]. Thus, it could be recommended that composite glucose- and fructose-based carbohydrates in a ratio close to unity are ingested irrespective of exercise duration. The suggestion that glucose-fructose mixtures be recommended (over single transportable carbohydrates) even when exercise duration lies within 1–2.5 h has the additional advantages of providing greater flexibility to increase carbohydrate intakes within an event if necessary.

Current recommendations do not advise athletes to ingest more than 90 g·h^−1^ of carbohydrates [1, 2] as it is believed that more carbohydrates do not offer additional benefits. Most studies to date investigating exogenous carbohydrate oxidation rates have been performed on moderately to highly trained athletes, but not in elite athletes whose absolute energy demands can be vastly higher. It has been reported that elite cyclists can tolerate more than 90 g·h^−1^ of carbohydrates mostly from glucose-based carbohydrates [70]. We have recently shown higher exogenous carbohydrate oxidation during prolonged endurance exercise in highly trained athletes with carbohydrate ingestion at 120 g·h^−1^ as compared to 90 g·h^−1^ [71]. However, endogenous carbohydrate oxidation was not further spared by the higher carbohydrate dose, and thus whether there would be an additional performance benefit requires clarification. Nonetheless, limited data support that carbohydrate intakes of up to 120 g·h^−1^ offer some benefits over 90 g·h^−1^ regarding *post-exercise* markers of muscle damage and recovery [72, 73]. Thus, this indicates that in certain scenarios, higher carbohydrate intakes than the currently recommended 90 g·h^−1^ could be beneficial.

Current evidence indicates that glucose delivery to the active tissue (i.e., muscle) limits exogenous carbohydrates oxidation rates. Bypassing intestinal absorption by infusing glucose and maintaining glycaemia did not result in higher exogenous glucose oxidation rates [74], whereas infusing glucose to maintain hyperglycemia (i.e., 10 mmol·L^−1^) enabled muscles to utilize up to 2 g·min^−1^ of exogenous glucose [75]. This occurred without a reduction in muscle glycogen use, but rather with suppression of fat oxidation rates. As oral ingestion of very large amounts of glucose (i.e., ~ 2.7 g·min^−1^) during moderate-intensity exercise did not cause plasma glucose concentrations to rise substantially and the rate of glucose appearance in the systemic circulation peaked at ~ 1 g·min^−1^ [55], intestinal absorption and/or extraction of glucose by the liver are the most plausible factors limiting exogenous carbohydrate oxidation rates. However, there is a relationship between exogenous carbohydrate oxidation rates and ingestion rates, and evidence shows that ingestion rates higher than 90 g·h^−1^ are feasible and that this results in very high exogenous carbohydrate oxidation rates [66, 76–79]. Yet, evidence for recommending such high ingestion rates (i.e., > 90 g·h^−1^) remains inconclusive, as there appears to be no further improvement in performance [80], no sparing of endogenous carbohydrate stores [71] and, in some cases, aggressive carbohydrate feeding during exercise can result in increased muscle glycogen utilization [81, 82]. It could be that athletes whose energy turnover rates during exercise are very high and thus have a high glucose flux to muscle due to increased blood flow proportional to absolute exercise intensity [83] could benefit from carbohydrate ingestion rates that are higher than currently recommended, but this requires further evaluation. Additionally, it remains to be established what occurs at intensities in heavy and severe exercise intensity domains, as some evidence indicates that despite higher carbohydrate demands, exogenous carbohydrate oxidation rates do not further increase [84]. Nonetheless, at present, it cannot be recommended to alter the currently recommended maximal carbohydrate ingestion dose during endurance exercise (i.e., up to 90 g·h^−1^).

There have been many attempts to increase exogenous carbohydrate oxidation rates by co-ingestion of carbohydrates with other nutrients or nutritional supplements, a topic recently reviewed by Baur and Saunders [85]. There was no effect from co-ingestion with protein [86], calcium [87] or sucralose [88], use of different glucose polymers [89] or use of drinks with different osmolality [90], whereas effects of caffeine co-ingestion yielded mixed results [91, 92]. On the other hand, 4 weeks of probiotic supplementation [93] and carbohydrate co-ingestion with l-arginine [94] increased exogenous carbohydrate oxidation rates, albeit to a degree (< 0.1 g·min^−1^), with likely limited practical relevance. More recently, use of hydrogel-forming carbohydrate drinks and gels has been popularized, and while the majority of studies found this strategy not to affect exogenous carbohydrate oxidation rates [95, 96], a recent study found that there could be a positive effect when using solely monosaccharide-based carbohydrate solutions (i.e., free glucose, free fructose) as opposed to a combination of polysaccharides (i.e., maltodextrin) and monosaccharides (i.e., fructose) [97]. More research is required to better understand a potential utility of the hydrogel.

#### Environmental Factors

While a vast majority of the studies discussed in the present article are based on interventions conducted in thermoneutral conditions, athletes are commonly required to exercise in extreme environments (e.g., hypoxia and high temperatures). In recent years, advances have been made in understanding how these environmental stressors affect carbohydrate metabolism during exercise [98] and more work is expected to be undertaken in the upcoming years. Both exercise in hypoxia and in the heat reduce the workloads at which the boundaries between moderate/heavy and heavy/severe exercise intensity domains occur ([99, 100]), making it more difficult to interpret studies due to potentially different energy turnover (i.e., when comparing responses to the same relative exercise intensity) or different physiological responses (i.e., when comparing responses to the same absolute exercise intensity).

When comparing exercise at the same relative exercise intensity between hypoxia and normoxia, there appear to be no differences in substrate oxidation rates [101], whereas if the absolute intensity is matched, there is an increase in carbohydrate oxidation rates [102]. This is met with a reduced ability to oxidize exogenous carbohydrates during exercise at both the same absolute [103] and relative intensity [104] It was hypothesized that this is largely explained by reduced peripheral insulin sensitivity because of increased oxidative stress in hypoxia [105]. Interestingly, altitude acclimation (i.e., 22 days of sojourning at altitude) alleviated the reduced ability to oxidize exogenous carbohydrates during exercise [103]. Thus, it appears that sufficient pre-exercise carbohydrate intake plays a crucial role in sustaining intense exercise in hypoxia [106].

Exercise in the heat at the same absolute intensity is accompanied with increased rates of glycogenolysis and thus glycogen utilization in non-heat acclimatized individuals [107–109]. This effect is somewhat alleviated with heat acclimation [110], making another argument towards the importance of undertaking heat acclimation before competing in the heat [111]. Similarly, as with hypoxia, exogenous carbohydrate oxidation rates in non-heat acclimated athletes are reduced under heat stress [112]. The underlying mechanisms are not yet completely understood. Among potential mechanisms are reduced carbohydrate absorption due to redistribution of the blood flow from the intestines towards the skin to support evaporative heat loss or a reduction in gastric emptying, which may be exacerbated with hypohydration [113], and/or increased rates of glycogenolysis due to elevated muscle temperature [109, 114]. The latter could in turn result in accumulation of glucose within the cell and a reduced glucose gradient between muscle cells and blood [115, 116]. Of note is also the fact that under heat stress, athletes are exercising at a higher relative exercise intensity, which drives increased carbohydrate oxidation rates [99, 117]. It remains to be explored whether heat acclimation somehow alleviates reductions in exogenous carbohydrate oxidation rates in the heat. From the applied perspective, currently, the most important solution to circumvent this is combining glucose- and fructose-based carbohydrates so higher exogenous carbohydrate oxidation rates can be achieved than those seen with glucose alone [118].

### Post-Competition

The main aim of carbohydrate nutrition in the post-competition period is recovery of liver and muscle glycogen stores. This does not necessarily imply that carbohydrate intake needs to be such that repletion of glycogen stores always needs to be rapid given that the next exercise session might not require full glycogen stores (e.g., an easy training session), but rather it must be tailored to the goals of the upcoming training/competition schedule. In this section, optimization of carbohydrate intake for scenarios when the next high-intensity training session/competition demanding high carbohydrate availability is followed in < 36 h is discussed, whereas other scenarios will be discussed in the Sect. 3. It is believed that for a full repletion of muscle glycogen stores, 24–36 h [21] are required, whereas for the complete repletion of liver glycogen, 11–25 h are needed [119]. Current nutritional guidelines recommend athletes ingest moderate to high glycemic index carbohydrates as soon as possible at the rate of 1.0–1.2 g·kg^−1^BM·h^−1^ to optimize repletion of glycogen stores for the first 4 h, when rapid repletion of glycogen stores is required, after which a normal diet reflecting daily fuel needs, which may be up to 12 g·kg^−1^ BM, is recommended [1, 2, 120].

However, a close examination of the literature reveals that these guidelines are perhaps too simplistic, especially for elite athletes. Scrutiny of the evidence for the optimal dosage of carbohydrates to be ingested in the early hours of post-exercise recovery reveals that there is only one study available comparing 1.2 g·kg^−1^ BM·h^−1^ (i.e., “optimal”) with a higher dosage (i.e., 1.6 g·kg^−1^BM·h^−1^), and this has been performed in moderately trained individuals with a BM index of ~ 26 kg·m^−2^ [121]. While it is difficult to compare results between different studies given that different methodological approaches have been used, it appears that there is a good relationship between the dosage and the amount of muscle glycogen resynthesis spanning at least from 0 to 1.8 g·kg^−1^BM·h^−1^ [120]. Thus, given that there is also a relationship between training status and the capacity to store muscle glycogen [28], it could be hypothesized that, absorption permitting, higher ingestion rates would be favorable to elite athletes whose relative proportion of muscle mass is higher. More research is required to elucidate if this is the case.

In addition to this, an emerging topic within the post-exercise recovery period, with an aim to improve functional capabilities of athletes, is the type of carbohydrates ingested in recovery. Namely, advances have been made on the type of carbohydrates (i.e., type of monosaccharides) ingested in the post-exercise recovery period that challenge current guidelines. While there appears to be no benefit of ingesting multiple types of carbohydrates (i.e., fructose and glucose) for optimization of muscle glycogen storage [45, 122], the benefits appear to lie with the time course of liver glycogen replenishment that is quicker when composite carbohydrates are ingested (i.e., glucose-fructose and galactose-glucose mixtures) [43, 45, 123]. Advancing these data are studies showing that recovery of cycling exercise capacity is greater after ingestion of a combination of glucose-based carbohydrates and fructose as compared to glucose-based carbohydrates only [124, 125], likely because of higher carbohydrate availability within both liver and muscle glycogen pools. It has been hypothesized (but not established) that combining glucose with both galactose and fructose would result in more rapid replenishment of both glycogen pools [126].

**Fig. 1:**
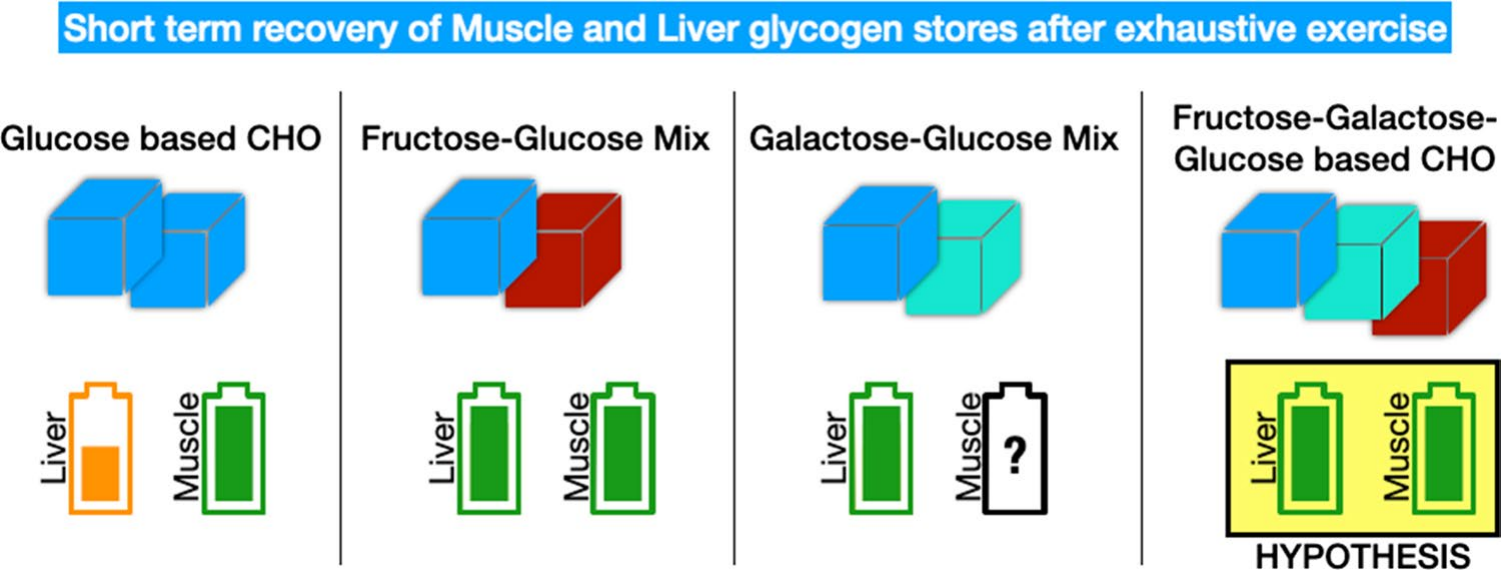
Short-term recovery of muscle and liver glycogen stores after exhaustive exercise using different combinations of monosaccharides. Fructose-glucose carbohydrate mixtures have been demonstrated to be very effective in replenishment of both muscle and liver glycogen stores. On the other hand, while glucose-based carbohydrates cause robust rates of muscle glycogen replenishment, liver glycogen synthesis rates are inferior as compared to a combination of fructose-glucose- and galactose-glucose-based carbohydrates. No data are currently available for muscle glycogen synthesis rates after ingesting a galactose-glucose mixture. It is hypothesized (but not established) that combining fructose-galactose-glucose-based carbohydrates would be optimal for post-exercise repletion of both glycogen pools. *CHO* carbohydrate

Interestingly, this strategy did not translate into improved cycling performance [127]. The results of the latter study are thus surprising. However, a close examination of the results offers a potential explanation and opens new research questions. Namely, two studies [125, 127] quantified utilization of in-recovery ingested carbohydrates in the subsequent exercise bout and found an increase in its use, indicating an increased carbohydrate availability. However, the increase of carbohydrate oxidation rates in the study assessing subsequent cycling performance was such that by the time the cycling time trial was initiated, glycogen stores within the body were likely the same in both conditions. Thus, more work is required to define the precise scenarios when a functional benefit can be expected; however, there appears to be a uniform observation that in terms of metabolism, ingestion of composite carbohydrates is beneficial. A summary of current knowledge on the effectiveness of different monosaccharide types on repletion of different glycogen depots (i.e., liver and muscle) is presented in Fig. 1. Based on the current evidence, it could be recommended that athletes seeking to recover glycogen stores as quickly as possible consider ingesting carbohydrates from a combination of glucose-based carbohydrates and fructose to optimally stimulate both liver and muscle glycogen resynthesis. The same recommendation cannot currently yet be given for galactose as whilst combined galactose-glucose favorably affects liver glycogen synthesis it is currently unknown how effective it is in the replenishment of muscle glycogen stores.

## Carbohydrates for Training

Training can be described as undertaking structured workouts with an aim to improve or maintain performance over time by manipulating the structure, intensity, duration and frequency of training sessions [128–130]. As total energy requirements and, consequently, carbohydrate demands are high in endurance-based sports, it is fair to assume that optimization of carbohydrate intake in these sport disciplines plays an important role. Early sports nutrition guidelines [131] advised athletes to both train and compete with high carbohydrate availability, and this approach dominated until 2005, when Hansen and colleagues observed that a reduction in carbohydrate availability before certain training sessions in untrained individuals could potentially enhance training adaptations [132]. In this study, leg kicking exercise training was performed in a 10-week-long training study. Each leg was subjected to a different treatment. Half of the training sessions in one leg were performed with reduced muscle glycogen content, while the other leg’s training sessions always commenced with normal glycogen stores. After the intervention, there was a substantial improvement in time to task failure at 90% of maximal workload in the leg that trained with reduced glycogen stores (+ 294%), while the increase in the other leg was much smaller (+ 125%). Muscle biopsy analysis also showed more positive metabolic adaptations (hydroxy acyl-CoA dehydrogenase [HAD] and citrate synthase [CS] activity) in the leg training with reduced muscle glycogen stores. While very attractive, the strategy was found to be effective in untrained individuals, and more work was required to see if similar findings could be observed in already trained individuals. As a result, this study was a landmark study paving the way for further investigations into whether different approaches to nutrient availability in trained athletes are beneficial based on different goals: training adaptation or competition performance.

In addition to carbohydrate availability manipulations to influence training adaptations, the concept of *training the gut* also needs to be considered to become a part of the training process to potentially improve tolerance to high carbohydrate ingestion rates during exercise especially [133, 134], as the prevalence of gastrointestinal issues during exercise is large [135, 136]. While the concept of training with high carbohydrate intakes to improve tolerance to ingested carbohydrates seems warranted, it remains to be established whether such practice leads to improved absorption of ingested carbohydrates and by what mechanisms or leads to just improved tolerance. Recent evidence from rats indicates that a combination of a high carbohydrate diet and exercise does not result in an increased number of glucose transporters in the intestines [137], and it could be thus speculated that improved tolerance can occur independently of improved absorption capacity.

### Carbohydrate Periodization

Building from the study by Hansen and colleagues, research started to focus on ways to optimize training adaptations and not necessarily optimize performance within these training sessions in trained individuals. Indeed, studies investigating molecular signaling responses after acute bouts of training with low muscle and liver glycogen stores in trained individuals provided promising results [10, 138]. As a result, the term *carbohydrate periodization* emerged whereby carbohydrate intake is adjusted daily based on the demands and/or goals of a certain training session. The concept is well described elsewhere [138, 139]. Using this approach, some studies demonstrated metabolic benefits, such as reduced reliance on carbohydrates during moderate-intensity exercise [140, 141]. However, a recent meta-analysis of nine studies investigating long-term benefits of carbohydrate periodization on performance outcomes suggests that this approach does not always enhance performance in the long term over training with high carbohydrate availability [142]. Perhaps important to understand when interpreting these data is that large training volumes are accompanied by substantial energy turnover. Even if a training session is initiated with adequate muscle glycogen stores, they will be markedly reduced by the end of it [28], creating a suitable environment for activation of crucial molecular signaling pathways thought to be responsible for positive adaptations [138].

One of the fundamental principles of endurance training is achieving sufficient training volume [143, 144]. For instance, elite cyclists are reported to cover more than 30,000 km on the bike in a single year [145]. Large training volumes are reported in other endurance sports as well [146]. This provides support for the notion that accumulation of sufficient training volume is of paramount importance among elite endurance athletes. Training with high carbohydrate availability (i.e., before and during) increases exercise capacity and performance, and inversely, training with low carbohydrate availability achieves the reverse effect, i.e., a reduction in exercise capacity [27, 53] and thus a reduction in training intensity and/or duration. Thus, training with low carbohydrate availability should likely be at best viewed as a more time efficient way to train [138, 147] rather than the optimal way. Commencing training sessions with insufficient carbohydrate availability would result in a reduction in the duration and/or intensity of the session, which are collectively the most important determinants of successful long-term endurance improvements [143]. However, as the athlete exhausts possibilities to improve performance by increasing training duration and/or intensity, reducing carbohydrate availability before training sessions could potentially be a useful strategy to increase the metabolic perturbation required to elicit training adaptations [148, 149] in a similar way to training in hypoxia or heat stress [150]. In line with this, a recent analysis demonstrated that both the degree of muscle glycogen utilization during exercise, which is mainly influenced by intensity/duration, and muscle glycogen concentrations at the end of an exercise bout, which could be influenced by carbohydrate availability pre- and during exercise, are important predictors of post-exercise 5ʹ adenosine monophosphate (AMP)-activated protein kinase (AMPK) activity [151]. Thus, manipulating carbohydrate availability before and during training sessions could affect molecular responses after exercise bouts. However, focusing solely on activation of pathways such as AMPK could be too reductionist, as it does not account for the recovery that is required after such a session, as, for instance, it is well known that protein breakdown is increased during such sessions [152, 153]. In addition to this, recent evidence indicates that the time between two exercise sessions rather than carbohydrate availability is the important modulator of the training responses after the second exercise bout [154, 155].

Collectively, the evidence indicates a limited utility of training with reduced carbohydrate availability most likely due to a decrease in training duration and/or intensity, unless one seeks the most time efficient training method. To circumvent this, attempts have been made to rescue the reduction in training capacity by utilization of ingestion of ergogenic aids. In line with this, carbohydrate and caffeine mouth rinsing have been shown to improve high-intensity exercise performance when conducted under a carbohydrate-restricted state [156]. Whether training adaptation can be enhanced with this approach has not been studied. More recently, building on previous work [157], the effects of delayed carbohydrate feeding in a glycogen depleted state (i.e., liver and muscle) on whole body metabolism and performance have been investigated [158]. While performance outcomes were unclear, delayed carbohydrate feeding enabled maintenance of stable blood glucose concentrations without suppressing fat oxidation rates and thus created a favorable metabolic response. Again, whether such an approach leads to longer-term enhancement in training adaptation remains to be seen. More broadly there is a need to further explore the potential benefits of commencing exercise with low carbohydrate availability to maximize both the metabolic and mechanical (i.e., exercise capacity) stimulus to optimize exercise adaptations.

### Training to Increase Fat Oxidation

Another popular reason for undertaking training with low carbohydrate availability is the notion that such an approach would lead to increases in fat oxidation rates during competition and spare endogenous carbohydrate stores with a limited storage capacity and by doing so improve performance [18, 159]. This view appears to be too simplistic, as numerous factors affect substrate utilization rates, including exercise intensity, training status, sex, acute and/or chronic diet [160, 161]. A recent study indicated that the capacity to utilize fat during exercise in an overnight fasted state is best correlated with CS activity [162], a marker of mitochondrial content [163] that is itself well correlated with training volume [144]. Thus, (1) the capacity to oxidize fat should naturally come together with improved training status and (2) specific manipulations of nutrition/training to alter fat oxidation rates during exercise should only be considered once the desired training status has been achieved. In other words, increasing fat oxidation rates as such should not come in front of the goal to improve one’s aerobic capacity, especially in the light of evidence demonstrating that training with low carbohydrate availability can result in a reduced ability to exercise at high intensities [164] due to reduced ability to utilize carbohydrates, as already discussed in previous sections [13]. More research is required to better understand if training and diet can be structured so that substrate oxidation rates would be altered in favor of fat oxidation without being part of general improvements seen with training per se, and whether this could lead to improvements in endurance performance.

### Low Carbohydrate Availability and RED-S

Unfortunately, the prevalence of relative energy deficiency in sport (RED-S) remains high [165]. Building on the previous evidence that sufficient carbohydrate intake can ameliorate symptoms of overtraining [166, 167], it has recently been proposed that there might be a link between relative RED-S and overtraining and that a common confounding factor is carbohydrate [11]. Recent data support an important role for dietary carbohydrate, as low carbohydrate, but not low energy availability, affects bone health markers [168], and deliberately inducing low carbohydrate availability to promote training adaptations and remaining in energy balance by increasing fat intake does not offer any benefits over a combination of energy and carbohydrate deficit—even more, it can impair glycemic regulation [169]. Whether carbohydrate availability is the crucial part in the development of RED-S remains to be properly elucidated.

### A Framework for Carbohydrate Periodization

**Fig. 2:**
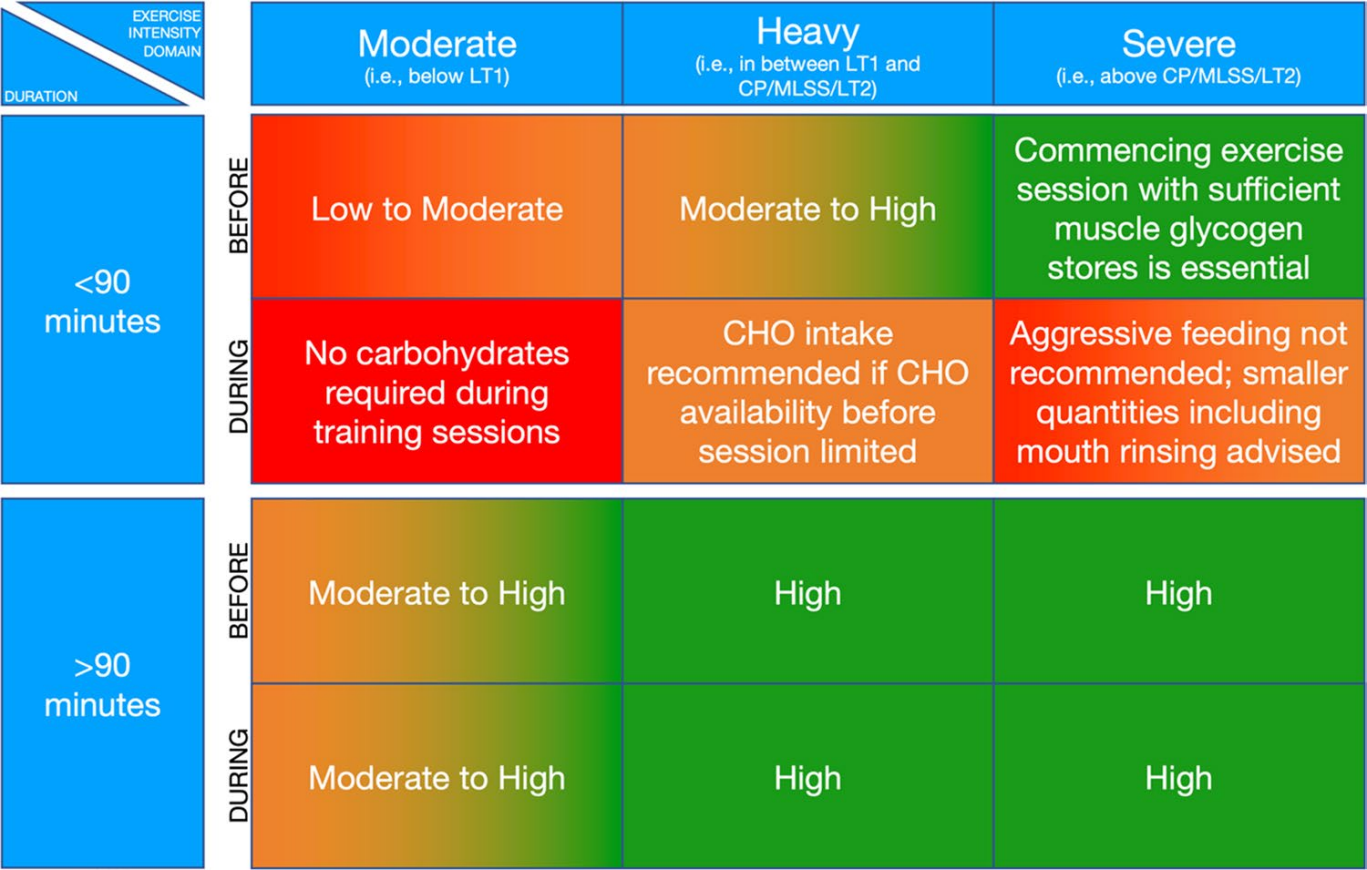
Framework for carbohydrate periodization based on the demands of the upcoming exercise session. Exercise intensity domain selection refers to the highest intensity attained during the exercise session. The exact carbohydrate requirements are to be personalized based on the expected energy demands of each exercise session. *CHO* carbohydrates, *CP* critical power, *LT1* lactate threshold 1, *LT2* lactate threshold 2, *MLSS* maximal lactate steady state

Collectively, periodizing carbohydrate intake based on the demands of training and especially an upcoming training session currently appears to be the most sensible approach as it (1) allows the execution of the prescribed training program, (2) minimizes the risk of high carbohydrate availability impeding training adaptations and (3) helps minimize the risk for occurrence of RED-S. A framework for carbohydrate periodization using this concept is depicted in Fig. 2.

While provision of exact recommendations for carbohydrate intake before and during exercise forms part of sports nutrition recommendations provided elsewhere [1, 2], we believe that interindividual differences in energy and thus carbohydrate requirements are such that optimization of carbohydrate intake should be personalized based on the demands and the goals of the exercise session one is preparing feeding for. For instance, aggressive provision of carbohydrate intake during exercise deemed beneficial among one population [73] in another population could lead to unwanted increase in muscle glycogen utilization [81]. In addition to this, even within sports commonly characterized as featuring extreme energy turnover rates, day-to-day differences are such that provision of exact carbohydrate guidelines would be too inaccurate [22, 170]. Thus, personalization of carbohydrate intake during exercise is warranted, as described in the next section.

## Personalizing Carbohydrate Intake

A certain level of personalization of energy and carbohydrate intake has been a standard part of nutritional guidelines for athletes for years [1, 2, 171]. Practitioners and athletes have a wide array of tools available that can help them personalize energy and carbohydrate intake. For instance, energy turnover for past training sessions and even energy requirements of the upcoming training sessions can relatively easily be predicted in sports where wearables exist to accurately quantify external work performed (i.e., power meters in cycling and running). Assuming fixed exercise efficiency one can then relatively accurately determine energy turnover during exercise. Knowing the relative exercise intensity of a given training session can further advance the understanding of the carbohydrate demands during exercise, as depicted in Fig. 2. As described in Sect. 2 of this article, it appears that absolute exercise intensity during exercise is a large determinant of total energy, and consequently carbohydrate [14] and exogenous carbohydrate oxidation rates [172, 173]. Thus, it is possible for athletes to predict energy turnover rates during exercise and adjust the carbohydrate intake accordingly.

In addition to this, the literature describing the physiological demands of a given sporting discipline can also be very insightful. For instance, energy turnover using gold-standard techniques has been assessed in many sporting contexts, including football [174], cycling [22] and tennis [175]. By knowing the energy demands, structure and goals of an upcoming training session, one can devise a suitable carbohydrate feeding strategy. Besides making predictions on total energy turnover during exercise, it is useful to establish the rate of glycogen breakdown, as very high-intensity efforts can substantially reduce muscle glycogen content without very high energy turnover rates [34, 176], especially as low glycogen availability can negatively affect performance [30]. Attempts have been made to find ways to non-invasively and cost-effectively measure muscle glycogen concentrations (e.g., using ultrasound); however, to date no independently performed validation has been successful in showing that this method of muscle glycogen quantification provides valid results [177]. Currently, the best solution is to draw on previous studies investigating glycogen breakdown by muscle biopsy in different sports disciplines, such as weightlifting [178], athletics [179], rugby [180], men’s football [181], women’s football [182] and Australian Rules football [183]. These data can be useful for practitioners to determine the relative (i.e., high, moderate or low) carbohydrate demands of a certain exercise session. However, whilst knowledge of exercise demands can help with tailoring, an implicit assumption is that all athletes will respond in a similar manner to an intervention, which may not be the case. In this respect, despite the present limitations in the practical assessment of muscle glycogen in field settings, gaining more readily accessible information on individual athlete physiological responses could still be of value to achieve higher degrees of personalization than those that current guidelines allow.

Recently, use of continuous glucose monitoring (CGM) devices has been popularized among endurance athletes, with an aim of personalizing carbohydrate intake around exercise for optimal performance. Certainly, knowledge of blood glucose profiles has the advantage that specific physiological data are generated from the individual athlete. These devices have a rich history in the field of diabetes treatment, and their utility has clearly been demonstrated [184]. For a device to be deemed of use and its use recommended to a wider audience, both of the following criteria must be met: (1) the parameter that the device is measuring should have contextual relevance (i.e., be providing useful information) and (2) the device must accurately measure what it is supposed to be measuring (i.e., be valid). While there is no doubt that CGM devices are useful in non-exercise contexts, their utility during exercise per se remains to be clearly established. Indeed, CGM devices appear to have limited validity during exercise [185, 186], and this may be due to the complex nature of blood glucose regulation during varying types and intensities of exercise. Blood glucose concentrations are a result of glucose uptake by the tissue and glucose appearance (i.e., liver glucose output and carbohydrate ingestion). While it has been known for a long time that hypoglycemia can associate with task failure [187], its occurrence does not always precede it [124]. This is especially the case during exercise of higher intensities where blood glucose concentrations increase [188] due to increased liver glucose output [20] and/or reduced muscle glucose uptake due to a reduced concentration gradient [116]. Therefore, further investigative work is required to establish whether differential blood glucose profiles using validated technology during exercise can be identified and be used to individualize carbohydrate intake during exercise.

In addition to tracking glycaemia during exercise, tracking it throughout the day could also be proven useful. A recent study utilizing CGM devices compared daily blood glucose profiles in elite trained athletes with those in a sedentary population and discovered large discrepancies in blood glucose concentrations throughout the day between both groups [189]. Elite athletes spent more time in hyper- and hypoglycemia as compared to sedentary controls, giving an appearance that glycemic control might be impaired. While periods of hyperglycemia are expected due to post-exercise high carbohydrate intakes, observations of hypoglycemia occurring especially at night during sleep were somewhat surprising. These data can be proven insightful if (1) it is established that these kinds of fluctuations have some negative effects on athletes’ function (e.g., recovery) and (2) a mechanistic basis of what is driving these changes is determined. This knowledge can then be used to potentially individualize strategies to counter these episodes of impaired glycemic control in real time.

While utilization of CGM devices during exercise to guide carbohydrate intake during exercise cannot be presently advised, athletes could individualize carbohydrate ingestion rates during exercise by establishing their highest exogenous carbohydrate oxidation rates [25]. To do this, one requires the ability to know carbon isotope enrichments of the ingested carbohydrates and in expired carbon dioxide. This approach requires specialized testing methodology that was once the preserve of the research environment but could become a standard part of athletes’ profiling alongside other measures typically made during metabolic testing (e.g., exercise economy or fuel utilization). For example, advances have been made in methodology to easier quantify stable carbon isotope abundance in expired air [190], a methodology currently used for quantification of exogenous carbohydrate oxidation rates [25]. Thus, this approach could be spun off from research and be used in practice as well to identify carbohydrate intake rate and carbohydrate compositions that optimize exogenous carbohydrate oxidation in individual athletes.

Finally, most research to date has investigated carbohydrate intake in a healthy male population, and thus current carbohydrate guidelines are founded on this evidence. However, there are some important metabolism differences between the sexes [191, 192] and/or relatively large populations of athletes with, e.g., diabetes type 1 [193] that would potentially benefit from more research work.

## Conclusions

Despite decades of intense carbohydrate research within the field of sports nutrition, new knowledge continues to be generated with the potential to inform practice. In this article, we have highlighted recent observations that provide a more contemporary understanding of the role of carbohydrate nutrition for athletes. For example, our article suggests a stronger emphasis be placed on scaling carbohydrate intake before competition to the demands of that subsequent activity, with particular attention paid to the effects of concomitant exercise during the preparatory period. Furthermore, athletes looking to benefit from carbohydrate feeding during exercise in the up to 60 g·h^−1^ range may benefit from a wide range of readily oxidizable sources, including glucose, glucose polymers and combinations of glucose-galactose (including lactose) or glucose-fructose (including sucrose), with the latter blend affording the greatest potential flexibility across a range of intakes. At high ingestion rates during exercise (i.e., > 60 g·h^−1^), glucose-fructose blends in ratios approaching unity (i.e., 1:0.8) appear preferable to the previously considered optimal ratio of 2:1. Furthermore, short-term recovery may be optimized by combining glucose-fructose to target both liver and muscle glycogen synthesis simultaneously. Finally, there has been substantial investigation into the role of commencing selected exercise sessions with reduced carbohydrate availability to provide a beneficial stimulus for training adaptation. However, a contemporary view of the “train-low” approach based on the totality of the current evidence suggests limited utility for enhancing performance benefits from training, but that its main benefit may lie in time efficiency.

The abovementioned suggestions are designed to build on the wealth of knowledge and recommendations already established for athletes. Nonetheless, what this review has also revealed is that gaps in our current understanding of carbohydrate nutrition and metabolism in relation to exercise performance remain. Some remaining research questions arising from the present article are presented in Table 1. Answering these research questions could allow continued advancement and refinement of carbohydrate intake guidelines and, by doing that, further increase the possibility of positively impacting athletic performance.
